# Trajectory of Body Mass Index and Frailty Among Older People in Southern Brazil: A Longitudinal Study

**DOI:** 10.3390/nu18020218

**Published:** 2026-01-09

**Authors:** Cecília F. Fernandes, Karla P. Machado, Andréa D. Bertoldi, Elaine Tomasi, Flávio Fernando Demarco, Maria Cristina Gonzalez, Renata M. Bielemann

**Affiliations:** 1 Graduate Program in Nutrition and Food, Federal University of Pelotas, Rua Gomes Carneiro 01, Pelotas 96010-610, Brazil; 2 Graduate Program in Epidemiology, Federal University of Pelotas, Rua Marechal Deodoro 1160, Pelotas 96020-220, Brazil; 3 Graduate Program in Dentistry, Federal University of Pelotas, Rua Gonçalves Chaves 457, Pelotas 96015-560, Brazil

**Keywords:** older adults, body mass index, frailty, trajectories

## Abstract

**Background/Objectives**: Frailty is a common geriatric syndrome associated with adverse outcomes such as disability, hospitalization, and mortality. This study aimed to assess the association between body mass index (BMI) trajectories over ten years and frailty among community-dwelling older adults in Brazil. **Methods**: This population-based longitudinal study used data from the COMO VAI? cohort, conducted with individuals aged ≥60 years in Pelotas, southern Brazil. Frailty was defined in 2024 using Fried’s phenotype, which evaluates weight loss, exhaustion, low physical activity, slowness, and weakness. BMI categories were defined as underweight (BMI < 22.0 kg/m^2^), eutrophy (22.0–27.0 kg/m^2^) and overweight (>27.0 kg/m^2^). BMI trajectories were identified using group-based trajectory modeling for 789 participants with data from at least two of three assessments (2014, 2019, 2024). Only trajectory groups comprising at least 5% of the sample were retained. Associations of baseline BMI and BMI trajectories with frailty were analyzed using Poisson regression with robust variance, adjusted for confounders and calf circumference. **Results**: Baseline underweight and overweight prevalence were 9.2% and 56.2%, respectively. Trajectory modeling identified three BMI groups: eutrophic (31.6%), overweight (56.4%), and obesity (12.0%). Obesity emerged as a distinct longitudinal trajectory rather than a baseline BMI category. Underweight did not emerge as a distinct BMI trajectory due to its low prevalence over time. Frailty prevalence in 2024 was 36.5%. Overweight trajectory participants had lower frailty prevalence after ten years (PR = 0.73; 95% CI: 0.54–0.99), while baseline underweight was associated with higher frailty ten years later (PR = 1.73; 95% CI: 1.05–2.84), consistent with the known risk of underweight and the potential protective effect observed in overweight older adults. **Conclusions**: Baseline underweight increased frailty risk, whereas an overweight trajectory showed a potential protective effect, consistent with the “obesity paradox” in older populations.

## 1. Introduction

Brazil is undergoing a rapid population aging process due to the demographic transition characterized by significant declines in fertility and mortality rates and increased life expectancy [1]. This trend aligns with global patterns, with estimates indicating that by 2050, the population aged 60 years and older will double, reaching about 2 billion people worldwide [2].

Aging is characterized by physiological changes that directly affect the health and nutritional status of older adults [3]. As people age, various changes occur, including shifts in body weight, often involving loss of muscle mass and increased body fat, especially in the abdominal area [4]. These changes in body composition are known to raise the risk of chronic diseases such as diabetes, hypertension, and cardiovascular conditions, and also reduce functional capacity in older adults [2]. These alterations are influenced by genetic, behavioral, and socioeconomic factors, which can either accelerate or slow the aging process [5].

In this context, body mass index (BMI) is a widely used anthropometric marker for estimating adiposity in older adults, reflecting the relationship between weight and height. Although it does not detect changes in body composition or exclude the presence of excess fat, undernutrition, or sarcopenia [6], BMI remains extensively used in population-based studies, where direct assessment of body composition is more challenging [7]. Despite its known limitations, it continues to be a practical and accessible tool for assessing nutritional health [8]. Given its widespread use and its strong association with nutritional status, BMI was selected as the main exposure to investigate long-term risk patterns for frailty in older adults. Additionally, longitudinal analysis of BMI enables investigation of the influence of demographic, socioeconomic, behavioral, and health-related characteristics on the nutritional trajectory of older individuals [9].

Long-term patterns of body mass index (BMI), rather than isolated measurements, provide important insight into the aging process. Persistently low BMI may reflect undernutrition, muscle mass loss, or underlying disease, whereas persistently high BMI is often associated with excess adiposity, metabolic dysregulation, and chronic inflammation. Both conditions can compromise physical function and physiological reserve over time. In older adults, these cumulative effects have been linked to adverse outcomes such as functional decline, cognitive impairment, increased hospitalization, mortality, and the development of frailty syndrome [10]. Frailty, the primary outcome of this study, is characterized by reduced resilience to stressors and manifests through clinical features such as unintentional weight loss, muscle weakness, slow gait speed, and exhaustion, several of which are closely related to nutritional status and long-term BMI patterns [11,12]. This highlights the relevance of BMI as a predictor of vulnerability in aging.

Some studies have indicated that changes in BMI over time, influenced by shifts in body composition, may contribute to the development of frailty, particularly in cases of undernutrition and obesity, which represent the extremes of nutritional status [13,14,15]. Recent evidence reinforces this relationship: a systematic review and meta-analysis of longitudinal studies demonstrated that both low and high BMI values, as well as their trajectories over time, are associated with an increased risk of frailty among older adults [16]. These conditions promote frailty through mechanisms such as chronic inflammation, sarcopenia, and metabolic alterations, negatively impacting functionality and quality of life [17,18]. Frailty has been strongly associated with an increased risk of all-cause and cause-specific mortality in older adults, as well as in younger adult populations [19].

Understanding this relationship is essential to expanding knowledge of the consequences of nutritional variations during aging, particularly in middle- and low-income countries such as Brazil. Therefore, the objective of this study was to evaluate the association between ten-year BMI trajectories and the occurrence of frailty among community-dwelling older Brazilian adults.

## 2. Materials and Methods

### 2.1. Design and Population

This longitudinal study uses data from the COMO VAI? study (Consortium for a Master’s-Oriented Program to Value Elderly Care), conducted with individuals aged 60 years or older residing in the urban area of Pelotas, southern Brazil. Data were collected at baseline in 2014 and in follow-up waves in 2019 and 2024. Participants included were community-dwelling older adults with sufficient cognitive capacity to respond to the questionnaire, or, if this was not possible, with the assistance of a caregiver or legal guardian. Participants were considered ineligible if they were unable to perform the required anthropometric measurements or physical tests due to physical or cognitive limitations.

**Figure 1:**
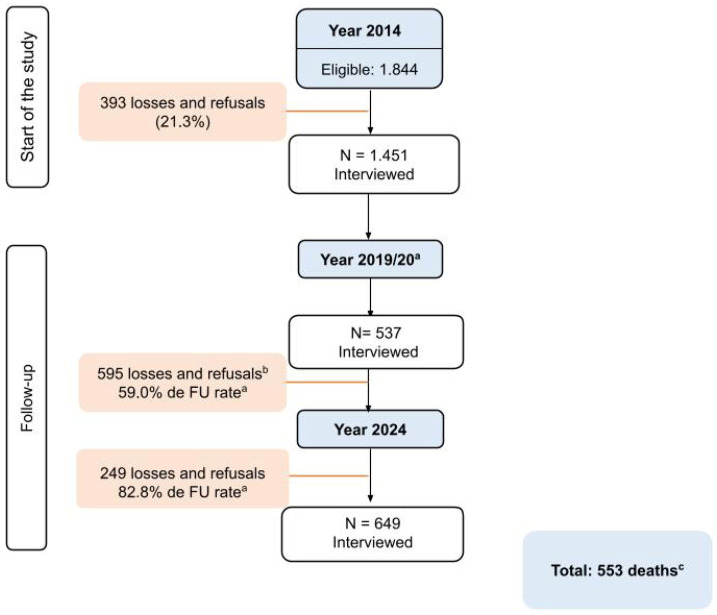
Flowchart of the COMO VAI? study. Notes: ^a^ FU rate, follow-up rate (excluding confirmed deaths). ^b^ The 2019 wave was interrupted due to the COVID-19 pandemic. ^c^ Deaths were identified through linkage with official municipal and state mortality records.

Figure 1 illustrates the flow of participants in the COMO VAI? study, from initial recruitment in 2014 to the follow-up waves in 2019 and 2024. The figure shows the number of eligible participants, losses and refusals, the follow-up rate (FU rate, excluding confirmed deaths), and deaths identified through official sources.

All study procedures were submitted for review and approved by the Institutional Research Ethics Committee under the following approval numbers: 472,357 (28 November 2013), 1,472,959 (31 March 2016), and 6,966,419 (25 July 2024), in accordance with the ethical principles established in Resolution No. 466 of 12 December 2012. Older participants and family members reporting deaths in person provided written informed consent at the time of the interview. The rights of participants to refuse participation or withdraw at any time, as well as their privacy, were ensured.

### 2.2. Sampling and Sample Size

Considering all research topics proposed in 2014 and accounting for potential losses and refusals, the sampling process was designed to include at least 1649 older adults, based on an expected outcome prevalence of 30%, a 95% confidence interval (CI), a four-percentage-point margin of error, and a design effect of 1.5 [20]. Sampling was conducted in two stages: first, 133 census tracts were selected based on the 2010 Census, and then households were randomly selected to identify at least 12 older adults per tract.

### 2.3. Data Collection

In all waves, trained interviewers collected data according to the methodological criteria of Habicht [21]. At baseline (2014), data were collected directly on netbooks. In the subsequent waves (2019 and 2024), information was recorded using tablets or smartphones through the electronic platform Research Electronic Data Capture (REDCap) (Vanderbilt University, Nashville, TN, USA) (https://projectredcap.org/).

### 2.4. Anthropometric Measurements and Body Mass Index

Body weight was measured in all waves using Tanita^®^ (Tokyo, Japan) electronic scales: model UM-080 in 2014 and model HS301 (digital and solar-powered) in the 2019 and 2024 waves, with a maximum capacity of 150 kg and precision to one decimal place. Height was assessed only in 2014, estimated from knee height. Knee height was measured with a wooden pediatric anthropometer (Indaiá^®^, Indaiatuba, Brazil), with participants seated, barefoot, and the knee flexed at 90°. Two measurements were taken, and if the difference exceeded one centimeter, a third measurement was performed. The mean of the two closest values was used. Height was then estimated using the predictive equations proposed by Chumlea and Guo [22], specific for both white and black individuals, of both sexes, aged 60 to 80 years. Body mass index (BMI) was calculated using the standard formula weight/(height)^2^, expressed in kg/m^2^ [23], and classified according to older adult-specific cut-off points [24]: underweight (BMI < 22.0 kg/m^2^), eutrophy (BMI 22.0–27.0 kg/m^2^), and overweight (BMI > 27.0 kg/m^2^), as recommended for individuals aged 60 years and older by the Brazilian Ministry of Health [25].

### 2.5. Outcome: Frailty

The frailty phenotype was assessed only in the 2024 follow-up wave and was defined by the presence of the following components: unintentional weight loss, weakness, slow gait speed, exhaustion, and low physical activity. Participants with none of these characteristics were classified as non-frail, those with one or two as pre-frail, and those with three or more as frail [12].

Weight loss was assessed by self-report with the question: “In the past 12 months, have you lost weight without dieting? If yes, how many kilograms?” A positive response indicating weight loss greater than 4.5 kg was considered as meeting this criterion [26]. Weakness was evaluated by measuring handgrip strength (kg) using digital hand dynamometers (Jamar Digital Plus+ Hand Dynamometer; Sammons Preston Canada, Mississauga, ON, Canada), with three measurements taken alternately on each hand, and the highest recorded value used. Measurements were performed with the participant seated on a sofa or chair with back support, knees flexed and together, feet flat on the floor, and elbow flexed at 90° with the wrist in a neutral position. Low muscle strength was defined as less than 29.7 kg for men and 16.2 kg for women, following the European Working Group on Sarcopenia in Older People (EWGSOP2) recommendations [27], using population-specific cut-off points equivalent to −2.5 standard deviations from the mean of a young reference population [28].

Gait speed was measured with a stopwatch by recording the time in seconds to walk a distance of four meters [29], and the shortest time from two trials was used. Low gait speed was defined as less than 0.8 m/s [27]. Exhaustion was assessed with two questions and considered present if participants answered “sometimes” or “most of the time” to at least one of the following: “In the past week, how often did you feel you could not carry out your tasks?” and “In the past week, how often did performing your routine activities require a great deal of effort?”

Physical activity was assessed using the leisure and transport sections of the International Physical Activity Questionnaire—Long Version (IPAQ), considering moderate- or vigorous-intensity activities lasting at least 10 min. Participants who did not meet the recommendation of at least 150 min of physical activity per week were considered inactive [30].

For analysis, frailty was divided into two groups: (1) frail and (2) non-frail/pre-frail, to facilitate interpretation and comparison of associations with other study variables, although the original three-category classification (non-frail, pre-frail, frail) was retained for descriptive purposes.

### 2.6. Covariates

Other covariates assessed at baseline in 2014 included demographic and socioeconomic characteristics: age (<75, ≥75 years), sex (male/female), skin color (White; Black/Brown/Yellow/Indigenous), marital status (married or with partner; separated/divorced or without partner; widowed), economic class according to the Brazilian Economic Classification Criteria by the Brazilian Association of Research Companies—ABEP [31], categorized as A/B, C, and D/E, and educational level in completed years (none, 1–7 years, ≥8 years). Behavioral variables included leisure-time physical activity according to the IPAQ (active/inactive, based on ≥150 min per week), smoking status (current smoker, former smoker, never smoker), and alcohol consumption in the last 30 days (yes/no). Health-related variables were also assessed. The number of chronic conditions was calculated based on self-reported medical diagnoses or the presence of the following conditions: hypertension, diabetes, heart problems, heart failure, asthma, bronchitis, emphysema, arthritis, Parkinson’s disease, kidney failure, hypercholesterolemia, seizures, stomach ulcer, osteoporosis, urinary incontinence, constipation, fecal incontinence, depression, glaucoma, hearing loss, difficulty swallowing, insomnia, fainting, rhinitis, difficulty speaking, stroke, mental disorders, and cancer. For analysis, a cut-off of five or more chronic conditions was used [32].

Calf circumference (CC) measured in 2014 was used as a marker of muscle mass [27]. The mean of two measurements of the right calf at its largest circumference was used, following the method described by Lohman et al. [33]. Low calf circumference was defined as ≤34 cm for men and ≤33 cm for women [34].

### 2.7. Statistical Analysis

Initially, a descriptive analysis of the outcome, exposure, and covariate variables was conducted. Categorical variables were described using proportions with their respective 95% confidence intervals (CI). Differences in baseline sociodemographic, behavioral, and health characteristics according to frailty status assessed in 2024 were evaluated using Pearson’s chi-square test.

BMI trajectories over time (2014, 2019, and 2024) were identified using group-based trajectory modeling with the “traj” command in Stata/IC^®^ software [35]. Participants with data from at least two of the three assessment waves were eligible for inclusion, as group-based trajectory modeling applies maximum likelihood estimation to handle missing data, allowing the inclusion of individuals with one missing follow-up. Consequently, the number of participants included in the trajectory analysis may differ from those with complete data in specific follow-up waves.

BMI trajectory groups represent longitudinal patterns of BMI change over time rather than fixed BMI categories at each assessment. Therefore, individuals were not required to remain in the same BMI category across the three waves. Participants who changed BMI categories over time were included in the analysis and assigned to the trajectory group for which they had the highest posterior probability of membership.

Zero-inflated Poisson (ZIP) and censored normal (cnorm) distributions were selected based on the nature of the variables. The number and shape of the trajectories were determined using the Bayesian Information Criterion (BIC), with the lowest value (regardless of sign) and the interpretability of the trajectories considered optimal [36]. Only trajectory groups with more than 5% of participants were included. Model selection also relied on average posterior probability scores (AvePP), which were required to exceed 0.7 for each group [36].

For participants with only two BMI measurements, posterior probabilities of trajectory membership were estimated using maximum likelihood based on their observed BMI values and the overall trajectory shapes derived from the full sample. Thus, even when BMI category changed between waves, individuals were classified into the trajectory group that best fit their longitudinal BMI pattern, according to the highest posterior probability.

Subsequently, the association between exposure variables and the frailty phenotype was evaluated using Poisson regression with robust variance to estimate prevalence ratios (PRs) and corresponding 95% confidence intervals (CIs). Non-frail participants were used as the reference group.

The main exposures were the BMI trajectories over time, as described above, and the BMI categories in 2014. Adjustment variables were organized into three hierarchical levels: the first level included sociodemographic characteristics (sex, age, skin color, marital status, education, and socioeconomic status); the second level included behavioral variables (leisure-time physical activity, smoking, and alcohol consumption); and the third level included health-related characteristics (number of chronic conditions, continuous BMI, and low calf circumference). Only variables with a *p*-value < 0.20 at each level were included in the model. Analyses using BMI trajectories as exposures were additionally adjusted for baseline BMI and included calf circumference as a marker of muscle mass. In analyses considering BMI in 2014 as the exposure, the adjustment model included the same hierarchical variables and also incorporated calf circumference as a muscle mass marker. Statistical significance was set at 5%, and all analyses were conducted using Stata/IC, version 17.0 (StataCorp, College Station, TX, USA).

## 3. Results

In 2014, a total of 1451 older adults were assessed at baseline in the COMO VAI? study. Of these, 537 participants were reassessed in 2019, a phase that was interrupted in March 2020 due to the COVID-19 pandemic. In the wave conducted in 2024, 649 older adults were interviewed. By September 2025, 553 deaths had been confirmed and 249 participants were classified as losses or refusals, resulting in an overall follow-up rate of 82.8% among participants in the COMO VAI? study.

Table 1 presents baseline sociodemographic, behavioral, and health characteristics of participants interviewed in 2024, according to frailty status assessed in that wave. At baseline, most participants were women, aged 60–74 years, self-identified as white, were married or partnered, and had less than eight years of education. Over half were classified as overweight, and a substantial proportion reported four or more chronic conditions.

When stratified by frailty status in 2024, frailty was more frequent among women, older individuals, those with lower educational attainment, and participants in lower socioeconomic classes. A higher prevalence of frailty was also observed among physically inactive participants, those who did not report alcohol consumption, and individuals with a higher number of chronic conditions. No statistically significant differences were observed for marital status, smoking, or baseline BMI categories (Table 1).

**Figure 2:**
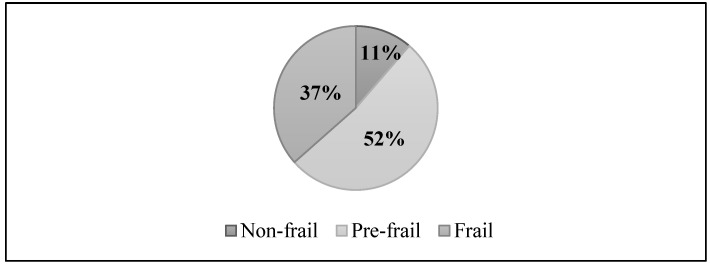
Classification of the frailty phenotype in 2024 among participants in the COMO VAI? study, Pelotas, RS, Brazil (n = 548). Note: Frailty classification according to Fried’s phenotype: participants with none of the five criteria (unintentional weight loss, exhaustion, weakness, slow walking speed, and low physical activity) were classified as non-frail; those with one or two criteria as pre-frail; and those with three or more criteria as frail.

The prevalence of frailty in 2024 was 36.5%. In analyses adjusted for baseline covariates, frailty was higher among women, older individuals (≥75 years: 61.2%), those with no formal education, and those in economic class D/E. Frailty was also more common among participants who were physically inactive, did not consume alcohol, and had a higher number of chronic conditions (≥7: 52.8%). Individuals classified as underweight in 2014 (45.7%) had a higher prevalence compared with those who were eutrophic (31.4%) and those who were overweight (38.7%). Figure 2 shows the distribution of the frailty phenotype in 2024, highlighting the predominance of pre-frail or frail individuals.

Before examining longitudinal BMI trajectories, we first analyzed the association between baseline BMI in 2014 and frailty assessed in 2024.

Table 2 shows the association between baseline BMI (2014) and the occurrence of frailty ten years later (2024). Individuals classified as underweight had a 73% higher prevalence of frailty compared with those with normal BMI (PR = 1.72; 95% CI: 1.10–2.70). This association remained statistically significant and of similar magnitude after further adjustment for low calf circumference (PR = 1.73; 95% CI: 1.05–2.84). Additionally, overweight participants had a 29% higher prevalence of frailty (PR = 1.29; 95% CI: 1.00–1.66) compared with those with normal BMI, although this association was no longer statistically significant after adjustment for low calf circumference.

**Figure 3:**
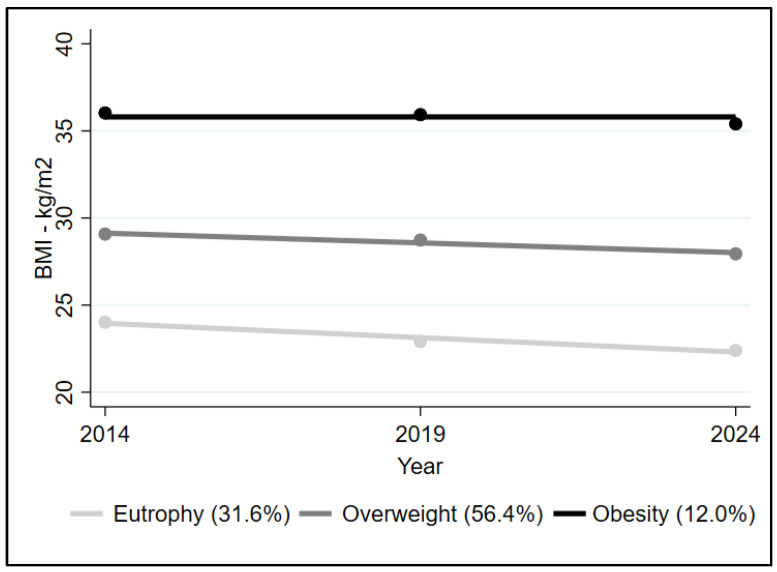
Trajectories of body mass index over 10 years (2014–2024) in participants of the COMO VAI? study, Pelotas, RS, Brazil (n = 789).

The group-based trajectory model classified BMI trajectories from 2014 to 2024 for 789 participants. Using this statistical approach, three trajectory groups were identified: two linear and one stable, with AvePP values of 0.90, 0.91, and 0.93, respectively. Trajectory group membership was determined based on longitudinal BMI patterns rather than fixed BMI cut-off points at each assessment. Based on the mean BMI of each trajectory over time, the groups were characterized as eutrophic, overweight, and obese, corresponding to 31.6% (n = 249), 56.4% (n = 445), and 12.0% (n = 95) of older adults, respectively (Figure 3). The normal BMI trajectory group had a mean BMI of 23.8 kg/m^2^ in 2014 and 22.2 kg/m^2^ in 2024; the overweight trajectory group had a mean BMI of 29.1 kg/m^2^ in 2014 and 27.9 kg/m^2^ in 2024; and the obesity trajectory group had a mean BMI of 36.2 kg/m^2^ in 2014 and 35.5 kg/m^2^ in 2024.

As shown in Table 3, a statistically significant association was found between the BMI trajectory of individuals classified in the overweight trajectory and the presence of frailty. In the analysis adjusted for baseline BMI, a 27% reduction in frailty prevalence in 2024 was observed among participants in the overweight trajectory (PR = 0.73; 95% CI: 0.54–0.99). This association persisted after further adjustment for low calf circumference (PR = 0.73; 95% CI: 0.53–1.00). Although the lower occurrence of frailty among participants in the obesity trajectory was in the same direction, it was not statistically significant.

## 4. Discussion

This study analyzed the association between BMI trajectories and baseline BMI with the occurrence of frailty among Brazilian older adults over a ten-year period. A high prevalence of frailty (36.5%) was observed, reinforcing its relevance as a major aging-related syndrome associated with increased mortality and adverse health outcomes [19]. Importantly, distinct patterns of BMI over time were differentially associated with frailty. Frailty was assessed using Fried’s phenotype, which includes unintentional weight loss, exhaustion, weakness, slow walking speed, and low physical activity, providing a multidimensional measure of vulnerability in older adults. While an overweight trajectory was associated with a lower prevalence of frailty at follow-up, baseline underweight emerged as a significant risk factor for frailty ten years later, highlighting the long-term impact of nutritional status on aging-related vulnerability.

Although this study is based on a population-based sample of older adults in Pelotas, Brazil, the findings may not be directly generalizable to older populations in different socioeconomic or ethnic contexts, particularly in high-income countries. Differences in lifestyle, healthcare access, nutritional patterns, and social support systems may influence both BMI trajectories and frailty development. Nevertheless, the observed associations provide relevant insights into the long-term impact of nutritional status on frailty, which could inform public health strategies in similar low- and middle-income settings.

The results of this study align with existing literature, which identifies underweight as a factor associated with increased risk of frailty, although the effects vary by age group, follow-up duration, and the definition of frailty used [16,37]. Meta-analyses and longitudinal studies have highlighted the protective effect of mild overweight in older adults, especially compared to underweight, which is linked to a higher risk of adverse outcomes such as frailty and mortality [38,39]. This relationship is consistent with what is described in the literature as the “obesity paradox,” in which mild or moderate excess weight in older adults, who are often affected by multiple chronic conditions, can provide metabolic reserves and protection against muscle and bone loss. Importantly, this finding reflects a statistical association rather than a clinical recommendation, as BMI does not differentiate between fat mass and muscle mass. Higher BMI values may reflect greater preservation of muscle mass, which could confer protection, or excess adiposity, which may increase risk of frailty, sarcopenia, malnutrition, and functional decline [27,40].

Furthermore, it is essential to consider the impact of survival on the observed results. Underweight is a well-recognized risk factor for mortality, and previous studies with the same cohort have shown a higher risk of death among older adults with this profile [41]. Thus, the methodological requirement to include only participants with at least two assessments, spaced by a minimum of five years, may have contributed to the absence of low-weight trajectory groups among those identified using the statistical approach. Consequently, the trajectories identified predominantly represent profiles with a higher likelihood of long-term survival. Even so, when analyzed individually, baseline underweight was associated with an increased risk of developing frailty. Using a similar methodological approach, a nine-year study with at least three BMI measurements classified participants into four BMI trajectories with a slight decline over time, without identifying low-weight trajectories, reinforcing the observation of early loss of these individuals during follow-up [42].

From a physiological and clinical perspective, malnutrition in older adults—of which underweight is one of the phenotypic criteria—is frequently associated with sarcopenia and chronic inflammatory processes. These conditions increase vulnerability and accelerate functional decline, contributing to greater frailty over time [43]. Additional mechanisms may also contribute to the association between BMI and frailty, including chronic low-grade inflammation, reduced metabolic reserve, and decreased functional resilience, which can affect muscle and fat tissue function, energy availability, and the capacity to recover from stressors. In this context, peripheral muscle mass, indirectly estimated by identifying low calf circumference and included in the adjusted models, helps explain the relationship between BMI and frailty observed in participants. It allows determination of whether higher BMI values reflect greater preservation of muscle mass, which is protective, or excess adiposity, which is associated with higher risk [27]. Individuals with low BMI are at higher risk of sarcopenia, malnutrition, and functional decline [44], conditions directly related to frailty. These findings highlight the need for continuous nutritional monitoring, with specific approaches to prevent frailty in underweight older adults, including those who previously had adequate weight.

Other studies have also examined the longitudinal association between BMI and frailty using various methodologies. For example, a study of 1125 older adults investigated the incidence of pre-frailty and frailty over two years, finding that underweight was associated with a higher risk of frailty [37]. A U.S. study identified four BMI trajectory classes—weight gain, weight loss, consistent obesity, and consistent overweight—and observed that changes in body weight were associated with the development of frailty, with an incidence of 19.9% over 10 years of follow-up [45]. The weight gain group had a higher likelihood of developing frailty (OR: 3.61; 95% CI: 2.39–5.46) compared to the consistent overweight group. The absence of a protective effect in trajectories with higher levels of adiposity, both in this and other studies [15], suggests that the benefits of overweight do not extend to obesity, possibly due to its impact on chronic inflammation and other comorbidities [46].

Considering that low CC is an indirect marker of low muscle mass, it was expected that including it in the models would attenuate the associations between BMI and frailty. However, the results remained virtually unchanged after adjustment, in contrast to findings from another study using the same sample [47], in which longitudinal reductions in CC over five years were associated with frailty and functional disability at the end of the period. This suggests that mechanisms other than muscle mass loss may contribute to the relationship between BMI and frailty.

This study has some limitations. First, caution is warranted regarding the findings related to the overweight trajectory group, as BMI, calculated from total body weight, does not differentiate body composition components or reflect adipose tissue distribution, despite statistical adjustment for another widely used anthropometric measure that serves as a proxy for muscle mass. Additionally, the COVID-19 pandemic affected data collection for the third wave, which began in 2019, resulting in fewer participants. However, comparative analyses showed that the profile of participants interviewed in 2019 did not differ significantly for most assessed characteristics, except for those known to be associated with higher mortality, such as older age [48]. Moreover, although changes in frailty prevalence over the 10-year period were observed, the study did not assess potential explanatory factors such as overall nutritional intake, changes in physical activity beyond leisure-time activity, or sociocultural influences. These unmeasured factors may have contributed to the observed trends, representing an additional limitation of the study.

Among the strengths, it is worth highlighting the long follow-up period, the high follow-up rate exceeding 80%, the objective assessment of the frailty phenotype using validated components, and the statistical consideration of various sociodemographic, behavioral, health-related, and muscle mass–related variables, which enabled better interpretation of the results. Furthermore, the application of BMI trajectory modeling contributes to methodological advances in understanding the relationships between longitudinal anthropometric profiles and frailty identification in aging.

## 5. Conclusions

In conclusion, this study found a high prevalence of frailty among older adults and highlighted the role of BMI over time as a factor associated with this outcome. Analysis of BMI trajectories showed that maintaining overweight status over the years was associated with a lower prevalence of frailty after ten years, consistent with the literature on the “obesity paradox.” Conversely, low body weight emerged as a risk factor for subsequent frailty. Importantly, these findings reflect statistical associations and should not be interpreted as a recommendation for weight gain. Rather, they underscore the need to prioritize nutritional health in older adults, with particular attention to preventing undernutrition and unintentional weight loss, as a strategy to preserve functionality, reduce frailty risk, and maintain quality of life in this population.

## Figures and Tables

**Table 1:** Sociodemographic, behavioral, and health characteristics at baseline (2014) among participants interviewed in 2024, according to frailty status assessed in 2024, in the COMO VAI? study, Pelotas, RS, Brazil.

Variables	Baseline (n = 1451)		2024 Wave (n = 649)		Prevalence of Frailty in 2024 (n = 548)		
	n (%)	CI 95%	n (%)	CI 95%	n (%)	CI 95%	*p*-Value
Sex (n = 1451)							0.002
Male	537 (37.0)	(34.6; 39.5)	216 (33.3)	(29.8; 37.0)	52 (27.7)	(21.7; 34.5)	
Female	914 (63.0)	(60.5; 65.4)	433 (66.7)	(63.0; 70.2)	148 (41.1)	(36.1; 46.3)	
Age (completed years) (n = 1451)							<0.001
60–74	1026 (70.7)	(68.3; 73.0)	560 (86.3)	(83.4; 88.7)	170 (34.1)	(30.0; 38.3)	
>75	425 (29.3)	(27.0; 31.7)	89 (13.7)	(11.3; 16.6)	30 (61.2)	(46.5; 74.1)	
Skin color (n = 1447)							0.014
White	1211 (83.7)	(81.7; 85.5)	545 (84.0)	(80.9; 86.6)	159 (34.3)	(30.1; 38.8)	
Black/Brown/Yellow/Indigenous	236 (16.3)	(14.5; 18.3)	104 (16.0)	(13.4; 19.1)	41 (48.2)	(37.6; 59.0)	
Marital status (n = 1447)							0.814
Married/With partner	763 (52.7)	(50.1; 55.3)	389 (59.9)	(56.1; 63.7)	123 (36.1)	(31.1; 41.3)	
Single/separated/divorced	225 (15.6)	(13.8; 17.5)	102 (15.7)	(13.1; 18.7)	30 (34.9)	(25.4; 46.1)	
Widowed	459 (31.7)	(29.4; 34.2)	158 (24.4)	(21.2; 27.8)	47 (38.8)	(30.5; 48.0)	
Economic level (n = 1372)							<0.001
A/B	483 (35.2)	(32.7; 37.8)	246 (40.1)	(36.3; 44.1)	56 (25.6)	(20.2; 32.0)	
C	720 (52.5)	(49.8; 55.1)	320 (52.2)	(48.2; 56.1)	112 (43.9)	(38.0; 50.1)	
D/E	169 (12.3)	(10.7; 14.2)	47 (7.7)	(5.8; 10.1)	22 (53.7)	(38.0; 69.0)	
Education (n = 1437)							<0.001
None	196 (13.6)	(12.0; 15.5)	63 (9.8)	(7.7; 12.4)	32 (66.7)	(52.0; 79.0)	
<8 years	782 (54.4)	(51.8; 57.0)	344 (53.5)	(49.6; 57.3)	126 (45.2)	(39.4; 51.1)	
>8 years	459 (32.0)	(29.6; 34.4)	236 (36.7)	(33.1; 40.5)	40 (18.5)	(14.0; 24.3)	
Leisure-time physical activity (n = 1391)							<0.001
Inactive	1133 (81.5)	(79.3; 83.4)	487 (76.3)	(72.9; 79.5)	163 (40.9)	(36.2; 46.0)	
Aticve	248 (18.5)	(16.6; 20.7)	151 (23.7)	(20.5; 27.1)	35 (25.0)	(18.5; 33.0)	
Smoking (n = 1446)							0.095
Non-smoker	781 (54.0)	(51.4; 56.6)	383 (59.0)	(55.2; 62.7)	119 (37.5)	(32.4; 43.0)	
Smoker	182 (12.6)	(11.0; 14.4)	62 (9.6)	(7.5; 12.1)	25 (47.2)	(34.0; 61.0)	
Ex-smoker	483 (33.4)	(31.0; 35.9)	204 (31.4)	(27.9; 35.1)	56 (31.5)	(25.0; 39.0)	
Alcohol intake (n = 1445)							<0.001
No	1138 (78.7)	(76.6; 80.8)	478 (73.6)	(70.1; 76.9)	163 (41.3)	(36.5; 46.2)	
Yes	307 (21.3)	(19.2; 23.4)	171 (26.4)	(23.1; 29.9)	37 (24.2)	(18.0; 32.0)	
Number of chronic conditions (n = 1339)							<0.001
<3	319 (23.8)	(21.6; 26.2)	158 (25.0)	(21.8; 28.6)	34 (23.6)	(17.3; 31.3)	
4 to 6	483 (36.1)	(33.5; 38.7)	248 (39.3)	(35.6; 43.2)	70 (32.7)	(27.0; 39.3)	
>7	537 (40.1)	(37.5; 42.8)	225 (35.7)	(32.0; 39.5)	93 (52.8)	(45.4; 60.2)	
Body mass index (n = 1364)							0.145
Underweight	126 (9.2)	(7.8; 10.9)	39 (6.1)	(4.5; 8.3)	16 (45.7)	(29.5; 63.0)	
Eutrophy	471 (34.5)	(32.0; 37.1)	201 (31.5)	(28.0; 35.2)	55 (31.4)	(25.0; 39.0)	
Overweight	767 (56.2)	(53.6; 58.8)	398 (62.4)	(58.5; 66.1)	128 (38.7)	(33.5; 44.1)	
Low calf circumference (n = 1369)							0.637
No	1032 (75.4)	(73.0; 77.6)	527 (82.0)	(78.8; 84.8)	162 (36.2)	(32.0; 41.0)	
Yes	337 (24.6)	(22.4; 26.9)	116 (18.0)	(15.2; 21.2)	38 (38.8)	(29.5; 49.0)	

Baseline data correspond to all participants interviewed in 2014 (n = 1451). The “2024 wave” column includes participants successfully followed up in 2024 (n = 649). Differences in sample size across columns reflect losses to follow-up. *p*-values were obtained using Pearson’s chi-square tests comparing frail and non-frail/pre-frail participants based on frailty status assessed in 2024.

**Table 2:** Association between nutritional status, assessed by body mass index, and the occurrence of frailty, according to crude analysis and adjusted models, COMO VAI? study, Pelotas, RS, Brazil (n = 519).

Frailty			
Initial BMI (2014)	Crude Analysis	Adjusted Analysis	
	PR (IC 95%)	PR (IC 95%) *	RP (IC 95%) **
Eutrophy	1	1	1
Underweight	1.46 (0.95; 2.22)	1.72 (1.10; 2.70)	1.73 (1.05; 2.84)
Excess weight ***	1.23 (0.95; 1.59)	1.29 (1.00; 1.66)	1.29 (0.98; 1.69)

* Model 1: adjusted for sex, age, education, marital status, alcohol consumption, leisure-time physical activity, and number of chronic conditions; ** Model 2: adjusted as in Model 1 + low calf circumference; *** Excess weight = overweight + obesity.

**Table 3:** Association between body mass index trajectories and the occurrence of frailty, according to crude analysis and adjusted models, COMO VAI? study, Pelotas, RS, Brazil (n = 513).

Frailty				
BMI Trajectories	Crude Analysis	Adjusted Analysis		
	PR (IC 95%)	PR (IC 95%) *	PR (IC 95%) **	PR (IC 95%) ***
Eutrophy	1	1	1	1
Overweight	0.88 (0.69; 1.13)	0.89 (0.70; 1.13)	0.73 (0.54; 0.99)	0.73 (0.53; 1.00)
Obesity	0.95 (0.66; 1.37)	0.92 (0.65; 1.31)	0.58 (0.32; 1.06)	0.58 (0.32; 1.06)

* Model 1 adjusted for sex, age, education, marital status, alcohol consumption, leisure-time physical activity, and number of chronic conditions; ** Model 2 adjusted for Model 1 + BMI measured in 2014; *** Model 3 adjusted for Model 2 + low calf circumference in 2014.

## Data Availability

The data presented in this study are not publicly available due to ethical and privacy restrictions involving human participants. De-identified data from the COMO VAI? cohort are available upon reasonable request and subject to approval by the study coordination team. Data access requires a collaboration agreement contributing to the continuity of the study. Requests should be addressed to comovaiufpel@gmail.com.
